# HLA‐G, IL‐6 and TGF‐β in Seminal Plasma as Potential Biomarkers of ART Outcome

**DOI:** 10.1111/aji.70195

**Published:** 2025-12-15

**Authors:** Andreas Schallmoser, Norah Emrich, Luca Masslow, Jean‐Pierre Allam, Rebekka Einenkel, Nicole Sänger

**Affiliations:** ^1^ Department of Gynecological Endocrinology and Reproductive Medicine University Hospital of Bonn Bonn Germany; ^2^ Department of Andrology University Hospital of Bonn Bonn Germany

## Abstract

**Background:**

The relationship between the immune system and embryo implantation is intricate and not yet fully understood. Although a genetically normal (euploid) embryo is essential, successful implantation also relies on carefully regulated immune responses that promote tolerance and prevent rejection. The main objective of this study was to examine the paternal levels of the immunoregulatory factors HLA‐G, transforming growth factor (TGF)‐ß and interleukin (IL)‐6 and to investigate possible correlations with other semen parameters, age and pregnancy outcome of the female partner.

**Methods:**

Seminal plasma samples from 225 men were collected from 2022 to 2025, divided into four groups (live birth, biochemical pregnancy, abortion, no pregnancy) and determined for immunological profiling using commercial HLA‐G, TGF‐ß and IL‐6 enzyme‐linked immunosorbent assay (ELISA) kits.

**Results:**

SHLA‐G levels were significantly lower in the live birth group compared to biochemical pregnancies (*p* < 0.001) non‐pregnancies (*p* < 0.001) and in abortions compared to non‐pregnancies (*p* = 0.004). TGF‐β levels were reduced in all pregnancy‐related groups versus non‐pregnancies (*p* < 0.001). IL‐6 levels were lower in biochemical pregnancies compared to non‐pregnancies (*p* = 0.017). Absolute HLA‐G levels were lower in live births (*p* < 0.001) and were reduced in abortions (*p* = 0.004) compared to non‐pregnancies. Absolute TGF‐ß values were significantly lower in the live birth group (*p* < 0.001) compared to non‐pregnancies. Absolute TGF‐β and IL‐6 levels were also decreased in biochemical pregnancies compared to non‐pregnancies (*p* < 0.001 and *p* = 0.03, respectively).

**Conclusion:**

Our findings show that seminal plasma levels of HLA‐G, TGF‐β and IL‐6 differ significantly among pregnancy outcome groups in ART cycles, underlining a potential role of these immunological factors in influencing reproductive success. These results highlight the importance of seminal immunological profiling as a possible predictive tool for ART outcomes and warrant further investigation in larger cohorts.

## 1 Introduction

Endometrial receptivity, embryo ploidy status and a synchronized cross talk between embryonic and maternal matrices are key necessities of successful embryo implantation [1, 2, 3, 4, 5, 6]. Evidence suggests that aneuploid embryos represent a major reason for abortion and implantation failure [7, 8, 9, 10, 11, 12, 13, 14, 15, 16, 17, 18].

However, selecting a euploid embryo results in a live birth of around 50%, drawing attention to the maternal immune system [19, 20], which may be influenced by paternal seminal plasma and potentially shift toward immunological tolerance [21]. Throughout decidualization, leukocytes are recruited to the uterus; during early pregnancy, the decidua consists of 40%–50% leukocytes. These leukocytes are necessary to support embryo implantation and the formation of the placenta. Simultaneously, the rejection of “foreign” antigens must be avoided by actively controlled tolerogenic mechanisms. About 70% of these leukocytes belong to the innate lymphoid cells (ILCs)‐subtype natural killer (NK) cells. Owing their name to their cytolytic characteristics systemically, NK cells show vastly differing features in the decidua. HLA mismatches in the classical HLA molecules between maternal immune system and the embryo, which could trigger rejection, are compensated by non‐classical HLA: HLA‐G and HLA‐E expressed by embryonic cells. Non‐classical HLA molecules are highly conserved compared to classical HLA molecules and exhibit an inhibitory effect on decidual cells suppressing their cytolytic activities and favouring the support of implantation rather than rejection. Thus, there is growing evidence that HLA‐G plays a prominent role in successful embryo implantation by causing local immunotolerance [22, 23] besides transforming growth factor beta (TGF‐ß).

Tolerance is only one of several aspects that the decidua must achieve. While it contributes to endometrial receptivity, selectivity is a separate and equally important function of the decidua. Numerous cytokines influence this balance and have been reported to play roles in both early and late pregnancy development. Consequently, recurrent implantation failure (RIF), which is hypothesized to result from a reduced receptivity‐to‐selectivity ratio, and recurrent pregnancy loss (RPL), which may arise from an elevated receptivity‐to‐selectivity ratio, are both influenced by these cytokine levels [24, 25]. This includes cytokines such as G‐CSF, IFN‐γ, IL‐1β, IL‐2, IL‐3, IL‐4, IL‐6, IL‐8, IL‐10, IL‐11 and LIF [26, 27, 28].

### 1.1 IL‐6

Interleukin‐6 (IL‐6) is a pro‐inflammatory cytokine involved in regulating immune responses, mediating inflammation and facilitating cell signalling [29]. Within the male reproductive tract, IL‐6 is secreted by various cell types, including macrophages and epithelial cells located in the testes, prostate and seminal vesicles [30]. While typically present at low concentrations in seminal plasma, IL‐6 levels can rise markedly in response to infections, inflammatory conditions or oxidative stress affecting the genital tract [31]. Increased levels of IL‐6 in semen have been linked to inflammatory disorders such as prostatitis, epididymitis and leukocytospermia, and are associated with detrimental effects on sperm quality. Given these associations, IL‐6 is increasingly recognized as a potential biomarker for detecting inflammation within the male reproductive system and for assessing its possible role in male infertility [31, 32, 33]. During implantation, a balanced amount of IL‐6 is necessary to support invasion of trophoblast cells and angiogenic processes. However, elevated IL‐6 levels, as expressed through excessive inflammatory responses to infections, represent a threat to implantation and pregnancy.

### 1.2 TGF‐ß

TGF‐ß is an important cytokine found in human semen. It plays a crucial role in regulating immune responses within both the male and female reproductive systems. Because of these functions, TGF‐ß has become a key subject of interest in the context of male fertility and reproductive immunology (34). TGF‐β coordinates to avoid rejection of paternal antigens of either sperm or expressed by the embryo. Throughout implantation and pregnancy, TGF‐β is released by various cell types in order to assure a tolerogenic micromilieu. TGF‐β is secreted by decidual stroma cell, leukocytes as well as trophoblast cells. Despite its tolerogenic characteristics, it also affects processes such as angiogenesis and trophoblast behaviour, thereby shaping implantation and formation of the placenta.

### 1.3 HLA‐G

Since the discovery of HLA‐G by Geraghty et al. in 1978 [35], studies from different fields such as cancer research, transplantation surgery and IVF have suggested HLA‐G to play a role not only in tumour tolerance [36, 37, 38] but also in immunomodulation at the foetal tolerance [39, 40, 41, 42]. The HLA‐G gene shows polymorphisms in coding and non‐coding regions that might influence HLA‐G transcription and translation on a molecular level and potentially regulates the amount of HLA‐G protein produced in any given cell or tissue. Anyway, the rate of polymorphisms seems to be low in humans, gorillas and chimpanzees; this points to a high evolutionary pressure [43]. Hypermethylation of the HLA‐G promoter [44] and decreased sHLA‐G levels in maternal blood have been suggested to play a role in pre‐eclampsia [45]. Several studies have indicated that sHLA‐G contributions from the developing embryo have an impact on pregnancy success [40, 46, 47, 48]. On the other hand, also in seminal fluid, different levels of sHLA‐G have been detected [41, 49].

### 1.4 Immunological Crosstalk at the Maternal–Foetal Interface Influenced by Paternal Antigens

Seminal fluid significantly affects cervical immune activity by stimulating the production of proinflammatory cytokines and attracting immune cells to the site [50]. In natural conception, seminal plasma is released into the vaginal cavity. It is suggested that a small amount of this plasma passes through the cervical mucus into the uterine cavity, aided by uterine peristaltic movements [51, 52, 53, 54], while certain components of seminal plasma may reach the uterus by attaching to the surface of spermatozoa [55, 56, 57].

## 2 Material and Methods

### 2.1 Ethical Approval

All patients were recruited at the Department of Gynecological Endocrinology and Reproductive Medicine, University Hospital of Bonn, Germany, from June 2022 until June 2025. All procedures performed in this study were in accordance with the Declaration of Helsinki and approved by the Ethics Committee of the University Hospital of Bonn, Germany (IRB00001791) approval code 369/21. Patients gave informed written consent.

### 2.2 Patients

Two hundred and fifty‐one seminal plasma samples from 225 couples were included in this study. The outcome of the subsequent ART procedure was matched to each sample. The chosen follow‐up time ensured a complete report of the live birth rate.

### 2.3 Semen Samples

Two hundred and fifty‐one semen samples from male donors were obtained between June 2022 and June 2025. Sperm preparation, assessment and analysis of semen samples were performed according to established protocols [41, 58]. In brief, semen samples were processed by separating sperm from seminal plasma using a density gradient system comprising of 1 mL of a 90% lower layer and of 1 mL of a 45% upper layer (Gynemed, Lensahn, Germany), overlaid with 1 mL of liquefied semen. The samples were centrifuged at 300 × g for 15 min. The resulting sperm pellet was transferred to a new tube and washed with 1 mL of sperm rinse medium (Vitrolife, Gothenburg, Sweden).

### 2.4 In Vitro Fertilization, Embryo Culture and Embryo Transfer

Before intracytoplasmic sperm injection (ICSI), enzymatic treatment was performed on cumulus oocyte complexes (COC) to remove cumulus cells. In case of IVF treatment, 100 000 motile sperm cells were added to up to three COC complexes. Pronuclear (PN) status was assessed 16–18 h after IVF/ICSI procedure according to Scott [59]; embryos were scored according to Gardner [14]. Single droplets of 25 µL GTL medium (Vitrolife, Gothenburg, Sweden) were used to perform embryo culture with oil overlay in dry flatbed incubators. Gas composition of in vitro atmosphere consisted of 6% CO_2_, 5% O_2_, and 89% N_2_ at 37°C.

### 2.5 Embryo Transfer and Pregnancy Definitions

Single embryo transfer procedures were predominantly performed on day 5. Assessment of serum hCG levels was conducted after 2 weeks. Clinical pregnancy rate was specified by the presence of a foetal heartbeat at 6–7 weeks of pregnancy. A biochemical pregnancy was defined as a serum hCG level ≥5 mIU/mL in the absence of a clinically confirmed pregnancy.

### 2.6 ELISA

HLA‐G, TGF‐ß and IL‐6 in seminal plasma were measured with commercially available ELISA Kits.

For HLA‐G assessment, the HLA‐G ELISA Kit Human (Aviva Systems Biology) was used. The ELISA was carried out according to the manufacturer's specifications with minor changes. Deviating from the manufacturer's protocol, the pre‐coated wells were blocked with 300 µL blocking buffer (0.05 M BSA in PBS pH 7.2) for 1 h at room temperature. Then, the manufacturer's protocol was followed. 1:50 000 diluted samples were used in the first measurement. If no signal was detectable, the 1:500 dilution of the same sample was measured in a second ELISA. ELISAs for TGF‐β (Human TGF‐beta 1 DuoSet ELISA) and IL‐6 (Human IL‐6 DuoSet ELISA; Bio‐Techne, Minneapolis, USA) were used according to the manufacturer's instructions. All samples were applied in duplicates. ELISA plates were measured with the TECAN Sunrise microplate reader. Optical density of each well was assessed at 450 nm with a wavelength correction at 540 nm. Standard curves and analysis were conducted by four parameter logistics using Quest Graph Four Parameter Logistic (4PL) Curve Calculator. AAT Bioquest, Inc., https://www.aatbio.com/tools/four‐parameter‐logistic‐4pl‐curve‐regression‐online‐calculator.

### 2.7 Statistics

Analyses of HLA‐G, IL‐6 and TGF‐ß values were performed for concentration (pg/mL) and total volume (pg) per semen sample (volume × concentration). Data was analysed using SPSS version 25 (IBM Armonk, New York, USA) with significance set at *p* < 0.05. Spearman correlation was used to analyse correlations between single parameters. A Kruskal–Wallis test was conducted to assess statistically significant differences between groups, followed by Dunn's post hoc test for pairwise comparisons.

Cytokine levels were expressed both as concentrations (pg/mL) and as absolute amounts per ejaculate (calculated as concentration × ejaculate volume). Values were therefore normalized to sample volume rather than total seminal plasma protein, to minimize variability associated with individual protein content. Group comparisons were performed using the non‐parametric Kruskal–Wallis test, and where significant, Dunn's post hoc test was applied for pairwise analyses. Correlation analyses between continuous variables were conducted using Spearman's rank correlation. Statistical significance was defined as two‐tailed *p* ≤ 0.05 after adjustment.

## 3 Results

No significant differences were observed among the groups in terms of male and female age, sperm concentration, male abstinence period or sperm volume as indicated in Table 1, while significant differences in immunological parameters were observed across groups with different pregnancy outcomes (Table 2).

**TABLE 1:** Baseline characteristics (median, min‐max).

Parameters	Live birth group *n* = 47	No pregnancy group *n* = 92	Biochemical pregnancy group *n* = 63	Abortion group *n* = 49	*p*
Male age (years)	36 28–49	37 28–48	37 28–50	36 27–54	0.465
Female age (years)	34 26–42	35 21–40	35 25–42	35 25–43	0.279
Male abstinence period (days)	5 2–21	5 2–14	4 2–24	5 2–14	0.909
Semen concentration (Mio/mL)	45.5 1–258	40.5 1–261	49.5 1–183	56 3–291	0.471
Semen volume (mL)	3 0.2‐7.5	3 1–7	3.0 1–8.2	3.9 1–7.3	0.705

*Note:* Kruskal–Wallis test‐.

**TABLE 2:** Parameters among different pregnancy outcome groups (median, min‐max).

Parameters	Live birth group *n* = 47	No pregnancy group *n* = 92	Biochemical pregnancy group *n* = 63	Abortion group *n* = 49	*p*
sHLA‐G (pg/mL)	0 0–959.2	166.7 0—495 202.1	44.02 0—680 676.9	0 0—55 364.8	**<0.001**
sHLA‐G (absolute)	0 0–4124.6	573.3 0–2 228 409.4	189.2 0–4 084 061.2	0 0—221 459.2	**<0.001**
TGF‐ß (pg/mL)	22 236.2 0–117 507.5	60 897.1 440.8–122 023.4	26 249.1 0–121 262.3	20 241.3 0–143 638.5	**<0.001**
TGF‐ß (absolute)	66 591.8 0–680,654.8	178 494.4 440.8–549 105.4	88 571.2 0–593,390.40	74 550.8 0–689,464.8	**<0.001**
IL‐6 (pg/mL)	0.8 0–35.6	3.0 0–1082.4	0 0–197.2	0.15 0–143.9	0.074
IL‐6 (absolute)	1.4 0–106.8	7.9 0–1948.2	0 0–591.5	0.76 0–705.1	0.096
Male age (years)	36 28–49	37 28–48	37 28–50	36 27–54	0.465
Female age (years)	34 26–42	35 21–40	35 25–42	35 25–43	0.279
Male abstinence period (days)	5 2–21	5 2–14	4 2–24	5 2–14	0.909
Semen concentration (Mio/mL)	45.5 1–258	40.5 1–261	49.5 1–183	56 3–291	0.471
Semen volume (mL)	3 0.2‐7.5	3 1–7	3.0 1–8.2	3.9 1–7.3	0.705

*Note:* Kruskal–Wallis test.

Bold values are statistically significant.

Using Dunn's post hoc test for group comparisons, sHLA‐G levels were significantly lower in live births compared to both biochemical pregnancies (*p* < 0.001) and non‐pregnancies (*p* < 0.001). Additionally, significantly lower sHLA‐G levels were observed in abortions compared to non‐pregnancies (*p* = 0.004).

TGF‐β levels were significantly lower in successful deliveries (*p* < 0.001), abortions (*p* < 0.001) and biochemical pregnancies (*p* < 0.001) compared to non‐pregnancies.

IL‐6 levels were significantly lower in the biochemical pregnancy group compared to the non‐pregnancy group (*p* = 0.017). Absolute sHLA‐G values were significantly lower in live births compared to biochemical pregnancies (*p* < 0.001) and the non‐pregnancy group (*p* < 0.001). Additionally, absolute sHLA‐G values were significantly decreased in the abortion group compared to the non‐pregnancy group (*p* = 0.004).

Absolute TGF‐β values were significantly lower in successful deliveries (*p* < 0.001), abortions (*p* = 0.002) and in the biochemical pregnancy group (*p* < 0.001) compared to the non‐pregnancy group.

Absolute IL‐6 values were significantly lower in the biochemical pregnancy group compared to the non‐pregnancy group (*p* = 0.03).

As indicated in Table 3, our analysis revealed significant positive correlations between TGF‐ß and semen concentration, between absolute TGF‐ß levels and abstinence time, and between HLA‐G and semen volume.

**TABLE 3:** Correlation of different parameters in seminal plasma.

	Male age (y) *p*	Concentration (10^6^/mL) *p*	Abstinence (d) *p*	Volume (mL) *p*	Motility (%) *p*
sHLA‐G (pg/mL)	0.404	0.847	0.834	**0.033**	0.090
sHLA‐G (absolute)	0.376	0.841	0.753	**0.003**	0.075
TGF‐ß (pg/mL)	0.769	**0.034**	0.234	0.878	0.712
TGF‐ß (absolute)	0.554	0.069	**0.019**	**<0.001**	0.357
IL‐6 (pg/mL)	0.754	0.735	0.278	0.073	0.753
IL‐6 (absolute)	0.849	0.827	0.196	0.906	0.665

*Note:* *n* = 251 seminal plasma samples Spearman correlation.

Bold values are statistically significant.

## 4 Discussion

Our study provides evidence that specific immunological components in seminal plasma, particularly sHLA‐G, TGF‐β and IL‐6, are differentially expressed among groups with varying pregnancy outcomes in ART cycles. These findings support the hypothesis that seminal plasma plays an active immunomodulatory role in early reproductive success, potentially influencing embryo implantation and tolerance induction in the female reproductive tract [41, 49, 60].

The biological relevance of HLA‐G in reproductive immunology lies in its central role in establishing maternal‐foetal immune tolerance. HLA‐G is a non‐classical class I major histocompatibility molecule expressed by extravillous trophoblast cells (EVTs) at the maternal‐foetal interface, where it interacts with inhibitory receptors (ILT2, ILT4 and KIR2DL4) on uterine natural killer (uNK) cells, macrophages and T cells. Through these interactions, HLA‐G suppresses cytotoxic responses, promotes regulatory T‐cell differentiation and supports vascular remodelling and placentation. Importantly, soluble HLA‐G (sHLA‐G) in seminal plasma may represent a paternal immunological signal that precedes trophoblastic HLA‐G expression. Exposure of the maternal reproductive tract to seminal sHLA‐G during intercourse could modulate local immune cells and induce a tolerogenic microenvironment even before embryo implantation. This pre‐conditioning may enhance immune adaptation to paternal antigens and facilitate subsequent recognition of trophoblast‐derived HLA‐G during early pregnancy. In this context, seminal and embryonic HLA‐G may act in a sequential, complementary manner, initiating and maintaining immune tolerance required for successful implantation and placental development [40, 42, 61, 62].

We found significant differences in sHLA‐G and TGF‐β levels between several outcome groups, particularly between biochemical pregnancies and live births and between abortions and non‐pregnancies, suggesting these markers may be involved in maternal immune adaptation processes essential for pregnancy progression. TGF‐β and specifically sHLA‐G as well as its absolute concentrations were significantly lower in the live birth group compared to biochemical pregnancies, abortions and non‐pregnancies.

The finding that lower seminal levels of sHLA‐G, TGF‐β and IL‐6 were associated with live birth outcomes appears counterintuitive given the well‐established immunosuppressive and tolerogenic functions of these molecules. However, elevated concentrations in non‐pregnancy or abortion groups may represent a compensatory but ineffective response to underlying immunological dysregulation. In such cases, excessive cytokine release could reflect an activated or proinflammatory seminal environment that impairs, rather than facilitates, the necessary immune adaptation within the female reproductive tract. Consequently, successful implantation and pregnancy may rely on a finely balanced immunological milieu, in which moderate levels of sHLA‐G and TGF‐β indicate a physiologically regulated seminal immune profile that supports appropriate maternal immune priming without inducing excessive tolerance.

TGF‐β and sHLA‐G in seminal plasma may enhance endometrial receptivity but simultaneously reduce endometrial selectivity, in line with the concept of the endometrium as a biosensor [25, 63]. Depending on the concentration of sHLA‐G, this modulation may either promote implantation through moderate levels or create an environment that is highly receptive but insufficiently selective, ultimately predisposing to early miscarriage. Given the pronounced interindividual fluctuations in sHLA‐G levels observed in this and previous work [41], this molecule appears capable of substantially influencing the receptivity–selectivity balance of the endometrium.

This interpretation aligns with the broader notion that both insufficient and excessive immunoregulation can be detrimental to implantation and placentation [62, 63]. Moreover, a nonlinear relationship may exist, wherein both very low and very high sHLA‐G concentrations negatively influence reproductive outcomes, reflecting the complex regulatory role of sHLA‐G in modulating NK and T‐cell activity locally in the uterus and systemically in maternal circulation [61]. It should also be noted that the extremely large variability in sHLA‐G concentrations, together with the chosen ELISA dilutions, allowed better resolution of high rather than low concentration ranges, which may have affected the detectability and differentiation of smaller concentration differences.

Similarly, TGF‐β, a well‐characterized immunosuppressive cytokine abundant in seminal plasma, showed significant differences across several outcome comparisons. TGF‐β was significantly higher in the non‐pregnancy group compared to those with live births, abortions and biochemical pregnancies. Given TGF‐β’s role in inducing regulatory T cells (Tregs) and promoting immune tolerance, our findings suggest that non optimal balanced seminal TGF‐β may impair the immunological crosstalk required for successful implantation and early pregnancy maintenance.

The significant difference of IL‐6 suggests it may also contribute to early pregnancy immunoregulation. IL‐6 is known to play dual roles, both pro‐ and anti‐inflammatory, depending on the microenvironment, and further research may clarify its nuanced function in ART settings.

Since treated by ART, the studied couples did not conceive naturally by sexual intercourse, which might bring up the question of the actual exposition of the endometrium to seminal plasma. Depending on the study, around 40% of the couples reported sexual intercourse before embryo transfer [64, 65]. In general, sexual intercourse throughout IVF treatment may increase biochemical and pregnancy rate depending on factors like timing. These effects might be explained by the regulating factors in seminal plasma, the exposition to paternal antigens as well as through stress‐reducing effects [66].

**FIGURE 1:**
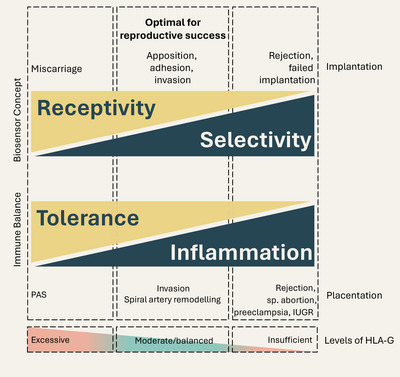
Summary and hypothesis. The concept of the endometrium as biosensor adapted from [63] combined with the immune balance required for implantation and placentation. Excessive TGF‐β and especially sHLA‐G amounts in seminal plasma may shift the balance towards foetal tolerance and receptivity but may result in elevated miscarriages. PAS, placenta accreta spectrum.

Despite initial implantation, the further invasion of trophoblast cells resulting in the formation of the placenta, equally requires a balanced immune micromilieu (see Figure 1). Foetal tolerance is necessary to avoid rejection of the semi‐allogenic or allogenic embryo (in case of egg donation or surrogacy), while embryonic and maternal cells are in direct contact. Controlled inflammatory cells and factors drive invasion and the spiral artery remodelling by supporting tissue remodelling processes. Moreover, invasion depth is directed by factors such as chemokines and limited by inflammatory and apoptotic signalling. Thus, an imbalance may result in abnormal invasion, remodelling capacities or even miscarriage by rejection. In case of HLA‐G, associations with pregnancy complications and adverse pregnancy outcomes are described. A decreased, insufficient level of HLA‐G was found in women with spontaneous abortions before the 12th week of gestation [22].

Genetic predispositions or other circumstances, which cause lowered HLA‐G expressions, may in this context also cause RPL. Moreover, in pre‐eclampsia, decreased levels HLA‐G expression are found. [67] Here, EVTs show improper invasion and insufficient spiral artery remodelling, which leads to changes throughout pregnancy. Shallow placentation may also result in intra‐uterine growth restriction (IUGR) as a result of insufficient HLA‐G levels. [68]. On the other side, increasing levels of HLA‐G are found at the implantation site of patients with placenta accrete spectrum. Here, it is hypothesized that the overly tolerogenic milieu fails to restrict the invasion of trophoblast cells.

Correlations between seminal immunological markers (Table 2) and semen parameters (Table 1) further support the biological relevance of these molecules. The association between TGF‐β and sperm concentration, HLA‐G and semen volume, and TGF‐β (absolute) with abstinence time may reflect the interplay between testicular/epididymal function and immune signalling, pointing towards systemic coordination of fertility‐related processes.

Collectively, these results suggest that the immunological composition of seminal plasma is not merely a passive by‐product, but potentially an important determinant of reproductive success, especially in the context of ART where normal sperm‐tract interactions may be bypassed. The inclusion of seminal plasma profiling in future ART protocols could provide new diagnostic or prognostic biomarkers and may even lead to adjunctive therapies, such as controlled seminal plasma exposure or immunomodulation prior to embryo transfer.

From a clinical perspective, seminal immunoprofiling could represent a valuable adjunct tool within assisted reproductive technology (ART). Measuring immunomodulatory factors such as HLA‐G, TGF‐β and IL‐6 may help identify male partners whose seminal immune environment is associated with suboptimal implantation or higher miscarriage risk. Such information could support patient stratification, guiding individualized ART management. For example, couples with dysregulated seminal cytokine patterns might benefit from targeted interventions‐such as anti‐inflammatory therapy, timing of intercourse before embryo transfer or controlled exposure to seminal plasma to induce maternal immune adaptation. These biomarkers could therefore become actionable early in the ART pathway, particularly at the stage of semen analysis or prior to oocyte retrieval and fertilization. Integrating immunological markers with standard semen parameters may enhance the predictive accuracy for ART outcomes and inform the design of personalized, immune‐aware reproductive strategies. Seminal immunoprofiling might complement the idea of uterine immunoprofiling (Lédée et al. 2017) and therefore provide an additional aspect of all the factors affecting implantation and early pregnancy development [24].

### 4.1 Limitations

The time between embryo transfer and the pregnancy test, as well as the choice of a threshold hCG value, result in a biologically unclear distinction between initial implantation and complete failure of implantation in the non‐pregnancy group. Therefore, significant differences in the non‐pregnancy group should also be interpreted with caution, as initial implantation cannot be fully excluded. Nonetheless, this distinction provides a clear definition of the ‘biochemical’ pregnancy group, which was the main focus of this project.

Although the total study cohort comprised 225 participants, the division into four clinical outcome groups resulted in comparatively small subgroup sizes. This may have reduced statistical power and could limit the generalizability of certain findings. The observed differences should therefore be interpreted with caution until confirmed in larger, independent cohorts. Nevertheless, the consistent trends across parameters and the robustness of the statistical analyses support the reliability of the observed associations.

In addition to age, several other factors may influence cytokine and immunomodulator levels in seminal plasma. These include subclinical genital tract infections, medication use, smoking, alcohol consumption, diet and body mass index (BMI). Such variables were not systematically recorded in the present study and could therefore act as potential confounders. Future research incorporating these clinical and lifestyle parameters, along with microbiological and metabolic profiling, will be important to disentangle their individual contributions to seminal immune signatures and reproductive outcomes.

The data analysis rather focused on capturing high concentrations of the assessed factors with a good resolution. Thus, rather high dilutions were chosen for ELISA with the aim to assess an overall broad range. Slight differences in low levels of the assessed factors, especially sHLA‐G, might be hidden in limited sensitivity, but were also not the focus of this study. Sperm morphology was not assessed, although it may play an important role in reproductive outcomes. The parity of the female partners was not recorded, which could represent a potential confounding factor. Additionally, although embryo transfers were performed predominantly on day 5, we did not analyse whether the timing of transfer influenced cytokine levels or clinical outcomes. Future studies should investigate whether the concentrations of HLA‐G, TGF‐ß or IL‐6 differ between day 3 and day 5 transfers. Furthermore, preimplantation genetic diagnosis (PGD) was not performed, and we therefore cannot account for embryo ploidy status, which may have affected implantation and pregnancy rates.

## Funding

Funding was provided by Theramex birth grant 2022.

## Conflicts of Interest

This study was financially supported by Theramex, with no conflict of interest arising from this support. The authors have no conflict of interest to disclose.

## Disclosure

All authors have approved the final article.

## Data Availability

The data that support the findings of this study are available from the corresponding author upon reasonable request.
